# GPER is involved in the stimulatory effects of aldosterone in breast cancer cells and breast tumor-derived endothelial cells

**DOI:** 10.18632/oncotarget.6475

**Published:** 2015-12-05

**Authors:** Damiano Cosimo Rigiracciolo, Andrea Scarpelli, Rosamaria Lappano, Assunta Pisano, Maria Francesca Santolla, Silvia Avino, Paola De Marco, Benedetta Bussolati, Marcello Maggiolini, Ernestina Marianna De Francesco

**Affiliations:** ^1^ Department of Pharmacy, Health and Nutritional Sciences, University of Calabria, Rende, Italy; ^2^ Department of Molecular Biotechnology and Health Sciences, University of Torino, Turin, Italy

**Keywords:** GPER, aldosterone, mineralcorticoid receptor, breast cancer cells, breast tumor-derived endothelial cells, Pathology Section

## Abstract

Aldosterone induces relevant effects binding to the mineralcorticoid receptor (MR), which acts as a ligand-gated transcription factor. Alternate mechanisms can mediate the action of aldosterone such as the activation of epidermal growth factor receptor (EGFR), MAPK/ERK, transcription factors and ion channels. The G-protein estrogen receptor (GPER) has been involved in the stimulatory effects of estrogenic signalling in breast cancer. GPER has been also shown to contribute to certain responses to aldosterone, however the role played by GPER and the molecular mechanisms implicated remain to be fully understood. Here, we evaluated the involvement of GPER in the stimulatory action exerted by aldosterone in breast cancer cells and breast tumor derived endothelial cells (B-TEC). Competition assays, gene expression and silencing studies, immunoblotting and immunofluorescence experiments, cell proliferation and migration were performed in order to provide novel insights into the role of GPER in the aldosterone-activated signalling. Our results demonstrate that aldosterone triggers the EGFR/ERK transduction pathway in a MR- and GPER-dependent manner. Aldosterone does not bind to GPER, it however induces the direct interaction between MR and GPER as well as between GPER and EGFR. Next, we ascertain that the up-regulation of the Na^+^/H^+^ exchanger-1 (NHE-1) induced by aldosterone involves MR and GPER. Biologically, both MR and GPER contribute to the proliferation and migration of breast and endothelial cancer cells mediated by NHE-1 upon aldosterone exposure. Our data further extend the current knowledge on the molecular mechanisms through which GPER may contribute to the stimulatory action elicited by aldosterone in breast cancer.

## INTRODUCTION

Aldosterone elicits multiple biological effects binding to the mineralcorticoid receptor (MR), which acts as a ligand-gated transcription factor [1]. In addition, rapid aldosterone signalling involves alternate mechanisms that include the activation of transduction pathways like tyrosine kinase c-Src, epidermal growth factor receptor (EGFR) and MAPK/ERK cascade [2-4]. Aldosterone is a key component of the renin-angiotensin-aldosterone system (RAAS), which is mainly implicated in maintaining salt and water balance toward the regulation of systemic blood pressure [5]. In addition, aldosterone activates ionic membrane transporters as the Na^+^/H^+^ exchanger (NHE-1) and Na^+^/HCO_3_^−^ cotransporter (NBC), which regulate the cellular pH and volume [6-7]. Aldosterone has been also involved in diverse cardio-metabolic diseases as it triggers inflammatory and fibrotic responses in both heart and vessels [8-11]. Recent studies have suggested that aldosterone/MR signalling may contribute to the progression of certain types of tumor [12-13]. For instance, it has been shown that aldosterone stimulates the survival and proliferation of renal carcinoma cells by upregulating K-RAS and the activation of the Akt and Raf pathways [12]. Moreover, an aldosterone blocker inhibited the growth of hepatocellular carcinoma and angiogenesis both *in vitro* and *in vivo* [13].

The G-protein estrogen receptor namely GPER mediates several pathophysiological functions in the cardiovascular, immune and central nervous systems, glucose and fat metabolism [14]. In addition, our and other previous studies have largely demonstrated that estrogenic GPER signalling elicits stimulatory effects in cancer cells and tumor microenvironment toward cancer progression [14-19]. In this regard, it has been reported that GPER activation triggers diverse transduction pathways involved in the proliferation, invasion and migration of tumor cells, including the epidermal growth factor receptor (EGFR), the MAPK/ERK and PI3K/AKT transduction cascades, Ca^2+^ mobilization and cAMP production [20-27]. Numerous endogenous, environmental and newly synthesized molecules have been shown to trigger relevant GPER-mediated responses in different cell contexts [28-36]. Aldosterone has been recently suggested to act through GPER in diverse models, including the cardiovascular and renal systems [6, 37-40]. For instance, it was demonstrated that GPER is involved in important effects exerted by aldosterone on vascular endothelial cells, cardiac vagal tone and connecting tubule glomerular feedback [37-40]. These observations have pointed out the potential of GPER to contribute to the aldosterone action, however the effective role played by GPER and the molecular mechanisms implicated are controversial as pharmacologic criteria for considering GPER as an aldosterone receptor have been not adequately fulfilled [41-43].

In the framework of the aforementioned observations, the current study provides novel insights into the role of GPER in mediating the action of aldosterone in breast tumor. In particular, our data show that a functional cross-talk between MR and GPER may occur upon aldosterone treatment leading to stimulatory effects in both breast cancer cells and endothelial cells obtained from breast malignancies.

## RESULTS

### Aldosterone activates the EGFR/ERK transduction pathway and induces the interaction between MR and GPER

**Figure 1:**
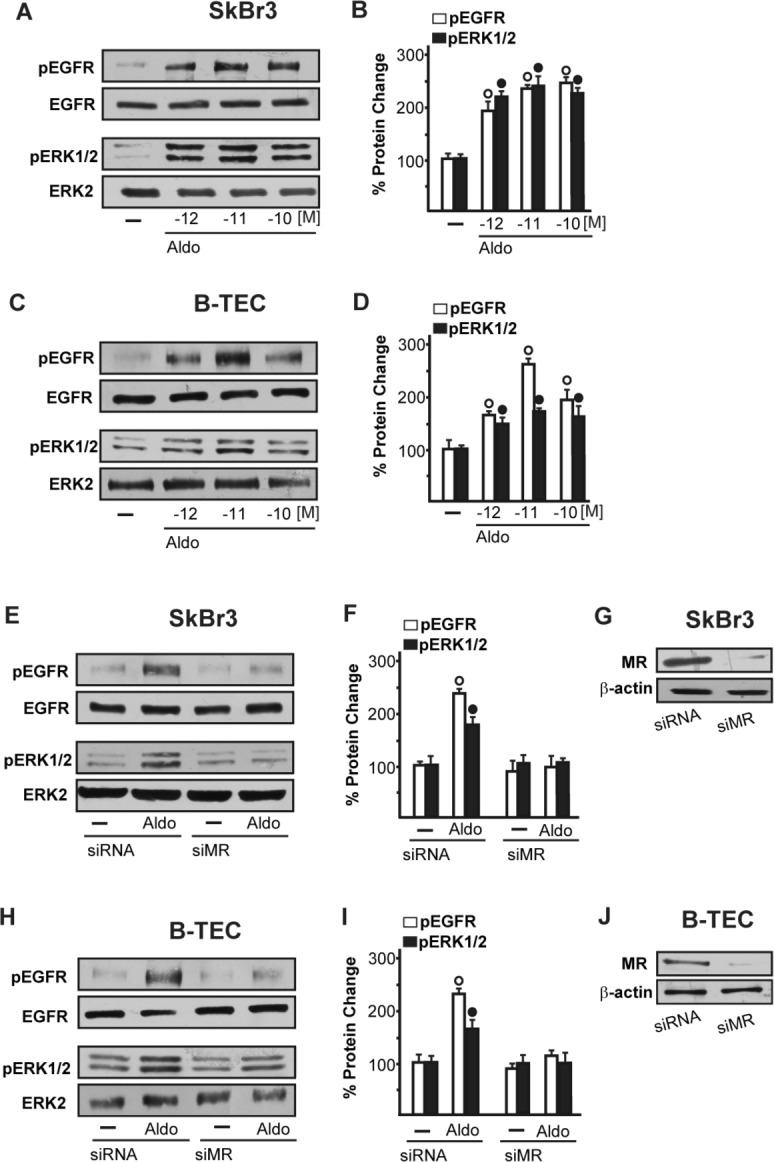
EGFR and ERK1/2 phosphorylation in SkBr3 cells **A.**, **B.** and B-TEC **C.**, **D.** treated with Aldosterone (Aldo) for 15 min. EGFR and ERK1/2 phosphorylation in SkBr3 cells **E.**, **F.** and B-TEC **H.**, **I.** transfected for 24 h with siRNA or siMR and then treated with 10 pM Aldo for 15 min. **G.**, **J.** Efficacy of MR silencing. The blots were normalized to EGFR or ERK2 and each data point represents the mean ± SD of three independent experiments. (○) and (●) indicate *p* < 0.05 for cells receiving vehicle (−) *versus* Aldo treatment.

**Figure 2:**
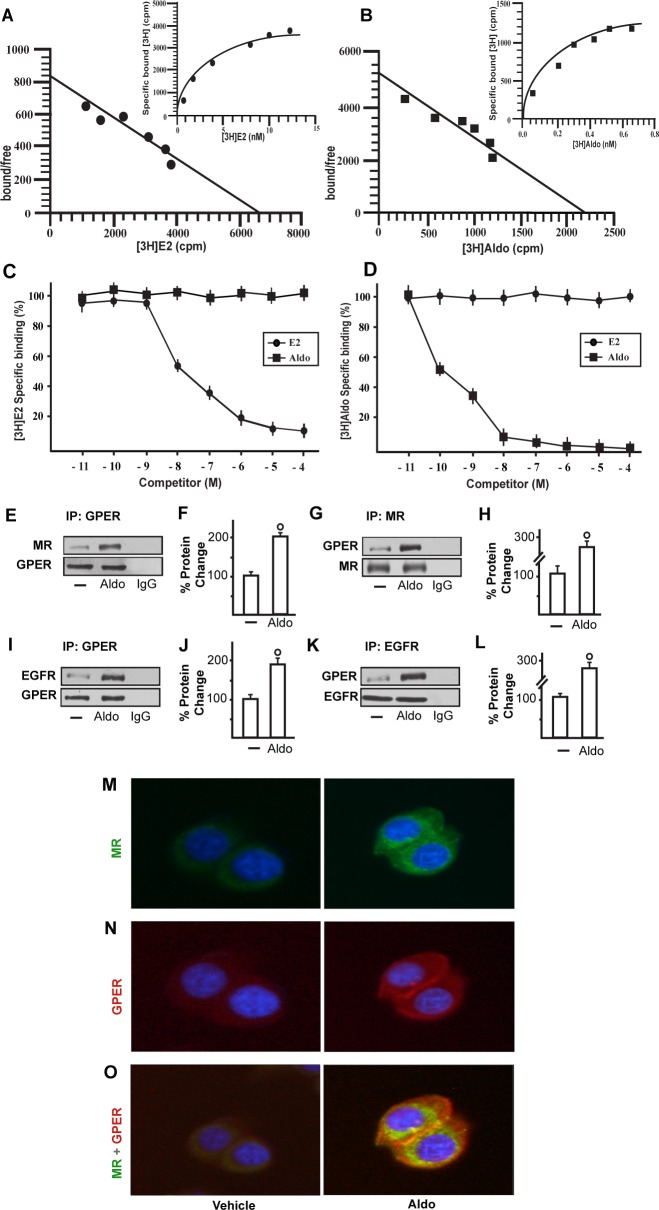
Representative saturation curve and Scatchard plot of [^3^H]17β-estradiol (E2) binding **A.** and [^3^H]Aldosterone (Aldo) binding **B.** in SkBr3 cells. Each value represents the mean ± SEM of three determinations. Ligand binding assay in SkBr3 cells incubated with [^3^H]E2 and exposed to increasing concentrations of E2 and Aldo for 2 hours **C.**. Ligand binding assay in SkBr3 cells incubated with [^3^H]Aldo and exposed to increasing concentrations of E2 and Aldo for 2 hours **D.**. Competition curves are expressed as a percentage of maximum specific [^3^H]E2 or [^3^H]Aldo binding. Each data point represents the mean ± SEM of three independent experiments performed in triplicate. The co-immunoprecipitation of MR with GPER increases upon treatment with 10 pM Aldo for 15 min in SkBr3 cells **E.**-**H.** The blots were normalized to GPER or MR, respectively. The interaction between GPER and EGFR increases upon treatment with 10 pM Aldo for 15 min in SkBr3 cells **I.**-**L.** The blots were normalized to GPER or EGFR, respectively. In control samples, nonspecific IgG was used instead of the primary antibody, as indicated. Each data point represents the mean ± SD of three independent experiments. (○) indicates *p* < 0.05 for cells receiving vehicle (−) *versus* Aldo treatment. Localization of MR **M.** and GPER **N.** alone or in combination **O.**, as evaluated by immunofluorescence in SkBr3 cells treated with 10 pM Aldo for 15 min. Green signal: MR; Red signal: GPER; Blue signal: Nuclei. Images shown are representative of ten random fields from three independent experiments.

We began our study evaluating whether aldosterone could be able to activate the EGFR/ERK transduction signalling in SkBr3 breast cancer cells and B-TEC breast tumor-derived endothelial cells, which were used as model systems. Both cell types express MR and GPER but not ERα (Supplementary Figure 1). Of note, pM aldosterone concentrations induced the phosphorylation of EGFR and ERK1/2 in both SkBr3 cells and B-TEC (Figure 1A-1D), though these effects were no longer evident silencing the expression of MR (Figure 1E-1J). Recently, it has been reported that GPER contributes to aldosterone action although the mechanisms involved remain to be fully understood [6, 38-44]. In this vein, we therefore performed saturation curves and scatchard plot analyses using as radiotracers the GPER ligand [^3^H]E2 [28, 31-32, 34-36] and the MR ligand [^3^H]aldosterone. [^3^H]E2 showed an estimated Bmax corresponding to 6799 ± 707.8 cpm/1 × 10^5^ SkBr3 cells and an estimated Kd corresponding to 8.16 ± 1.70 nM (Figure 2A), whereas [^3^H]aldosterone showed an estimated Bmax corresponding to 2159 ± 229.2 cpm/1 × 10^5^ SkBr3 cells and an estimated Kd corresponding to 0.42 ± 0.08 nM (Figure 2B). In competition assays, E2 but not aldosterone displaced [^3^H]E2 (Figure 2C), while aldosterone but not E2 displaced [^3^H]Aldosterone (Figure 2D). Collectively, these findings argue that in SkBr3 cells aldosterone is not able to displace [^3^H]E2, which was used as a GPER radioligand.

In order to gain further insights into the role of GPER in certain biological responses to aldosterone, we then evaluated the possible interaction of GPER and MR and EGFR. Our immunoprecipitation data indicated that aldosterone triggers a direct interaction between GPER and MR as well as GPER and EGFR (Figure 2E-2L). Immunofluorescence experiments performed in SkBr3 cells further corroborated the aforementioned results as an increased merged (orange) signal of MR and GPER was observed upon a short (15 min) aldosterone treatment (Figure 2M-2O). Altogether, these data suggest that GPER may contribute to aldosterone/MR-activated EGFR signalling.

### GPER is involved in the aldosterone-mediated signalling

**Figure 3:**
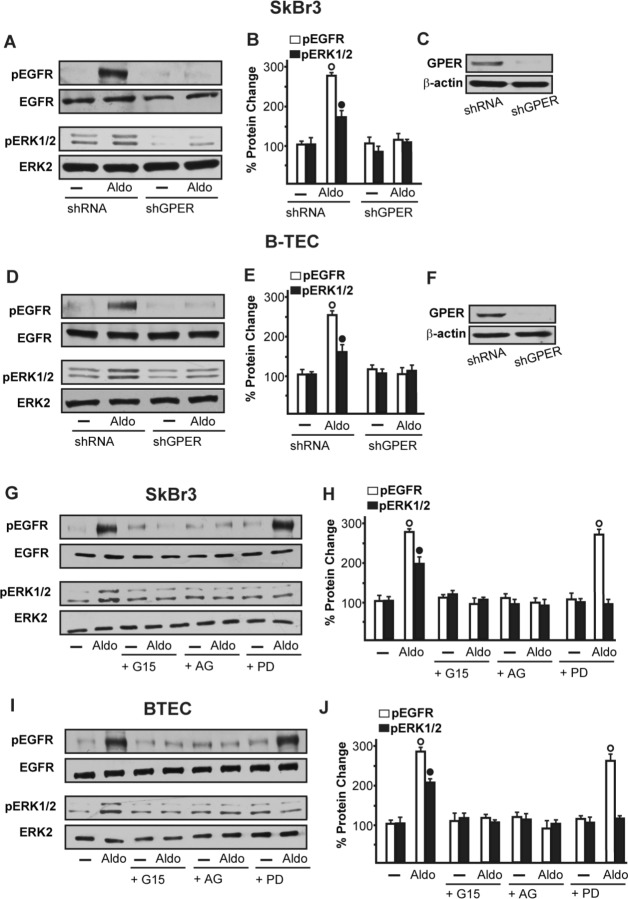
EGFR and ERK1/2 phosphorylation in SkBr3 cells **A.**, **B.** and B-TEC **D.**, **E.** transfected for 24 h with shRNA or shGPER and then treated with 10 pM Aldo for 15 min. **C.**, **F.** Efficacy of GPER silencing. EGFR and ERK1/2 activation in SkBr3 cells **G.**, **H.** and B-TEC **I.**, **J.** treated for 15 min with 10 pM Aldo alone and in combination with 10 μM EGFR inhibitor AG1478 (AG), 10 μM MEK inhibitor PD98059 (PD) and 100 nM GPER antagonist G15. The blots were normalized to EGFR or ERK2 and each data point represents the mean ± SD of three independent experiments. (○) and (●) indicate *p* < 0.05 for cells receiving vehicle (−) *versus* Aldo treatment.

**Figure 4:**
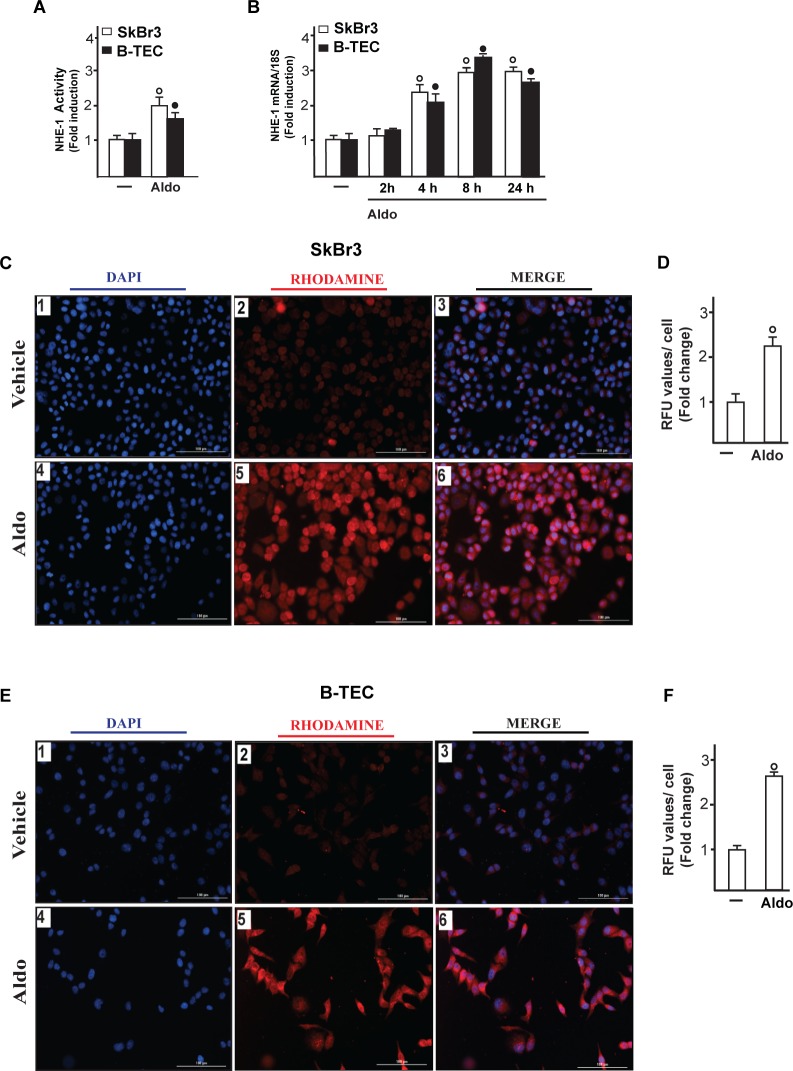
Na^+^/H^+^ Exchanger 1 (NHE-1) activity in SkBr3 cells and B-TEC treated with 10 pM Aldo, as evaluated by fluorescence intensity measurement **A**. Each data point represents the mean ± SD of three independent experiments. mRNA expression of NHE-1 in SkBr3 cells and B-TEC treated with 10 pM Aldo, as evaluated by real-time PCR **B.**. Values are normalized to the 18S expression and shown as fold changes of the mRNA expression induced by Aldo respect to cells treated with vehicle (−). NHE-1 expression as evaluated by immunofluorescence in SkBr3 cells **C.** and B-TEC **E.** treated with ethanol as vehicle or 10 pM Aldo for 8 hours. NHE-1 accumulation is shown by the red signal, nuclei were stained by DAPI (blue signal). Images shown are representative of three independent experiments. **D.**, **F.** Fluorescence intensities for the red channel were quantified in 10 random fields for each condition and results are expressed as fold change of relative fluorescence units (RFU) over the vehicle-treated cells. (○) and (●) indicate *p* < 0.05 for cells receiving vehicle (−) *versus* Aldo treatment.

**Figure 5:**
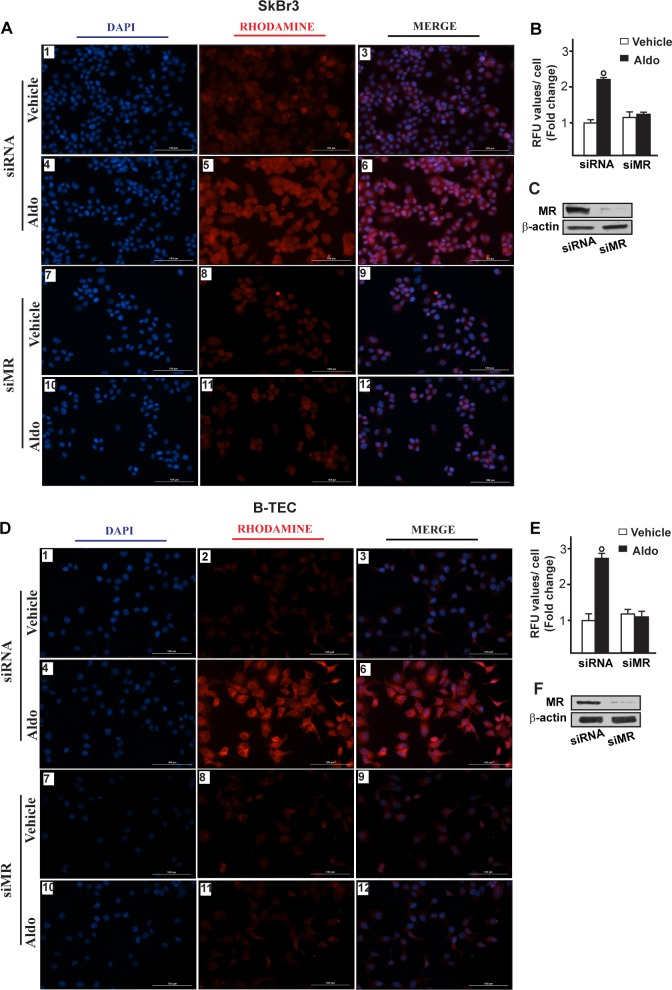
Na^+^/H^+^ Exchanger 1 (NHE-1) expression as evaluated by immunofluorescence in SkBr3 cells **A.** and B-TEC **D.** transfected for 24 hours with siRNA (panels 1-6) or siMR (panels 7-12) and then treated with ethanol as vehicle or 10 pM Aldosterone (Aldo) for 8 hours. NHE-1 accumulation is shown by the red signal, nuclei were stained by DAPI (blue signal). Images shown are representative of three independent experiments. **B.**, **E.** Fluorescence intensities for the red channel were quantified in 10 random fields for each condition and results are expressed as fold change of relative fluorescence units (RFU) over the vehicle-treated cells. **C.**, **F.** Efficacy of MR silencing. (○) indicates *p* < 0.05 for cells receiving vehicle *versus* Aldo treatment.

**Figure 6:**
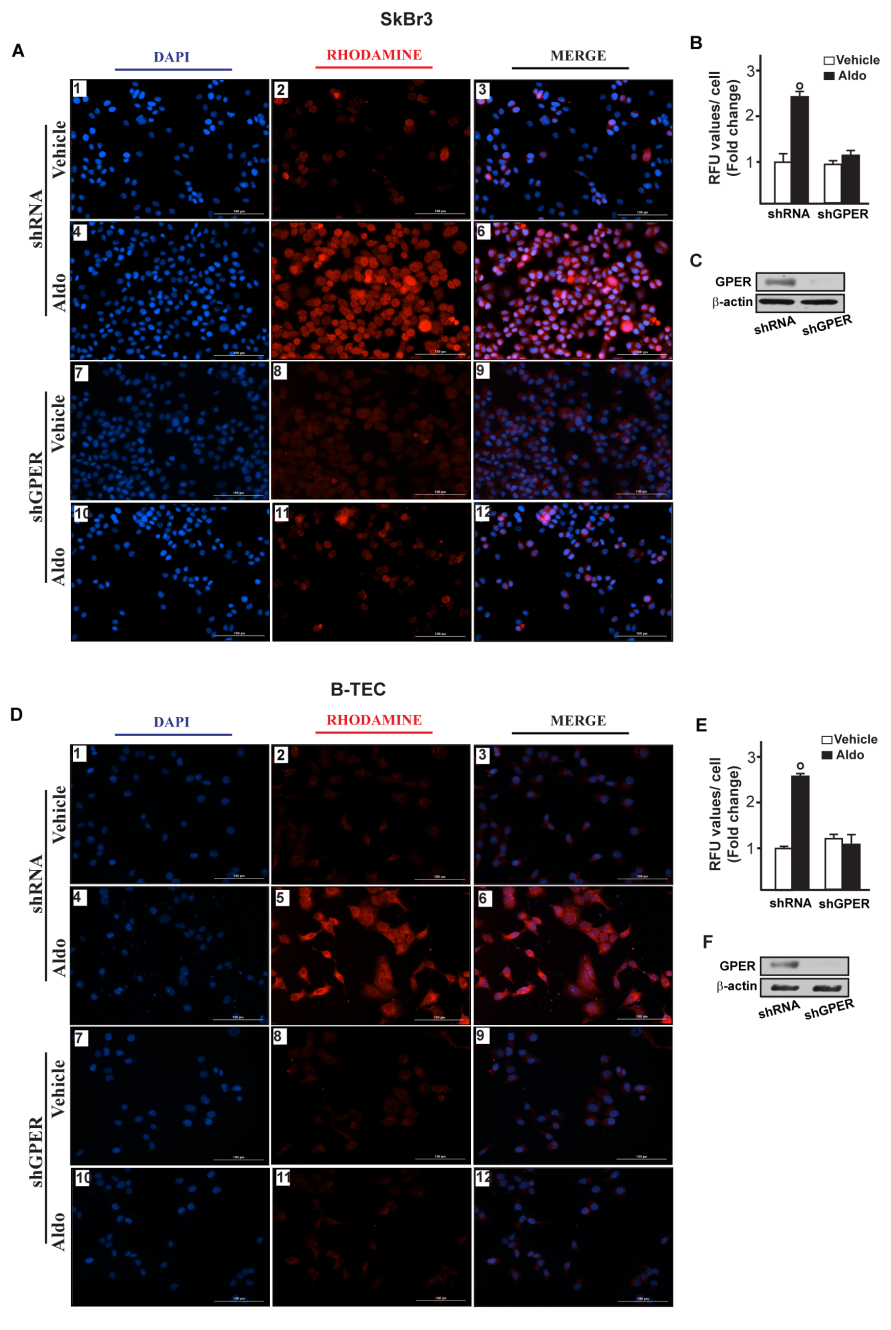
Na^+^/H^+^ Exchanger 1 (NHE-1) expression as evaluated by immunofluorescence in SkBr3 cells **A.** and B-TEC **D.** transfected for 24 hours with shRNA (panels 1-6) or shGPER (panels 7-12) and then treated with ethanol as vehicle or 10 pM aldosterone (Aldo) for 8 hours. NHE-1 accumulation is shown by the red signal, nuclei were stained by DAPI (blue signal). Images shown are representative of three independent experiments. **B.**, **E.** Fluorescence intensities for the red channel were quantified in 10 random fields for each condition and results are expressed as fold change of relative fluorescence units (RFU) over the vehicle-treated cells. **C.**, **F.** Efficacy of GPER silencing. (○) indicates *p* < 0.05 for cells receiving vehicle (−) *versus* Aldo treatment.

On the basis of the abovementioned observations, we performed gene silencing experiments in order to assess whether GPER is involved in the rapid signalling induced by aldosterone. Interestingly, the activation of both EGFR and ERK1/2 by aldosterone was no longer evident silencing GPER in both SkBr3 cells and B-TEC (Figure 3A-3F). In accordance with these findings, the GPER antagonist G15 prevented the EGFR/ERK phosphorylation upon aldosterone exposure (Figure 3G-3I). Next, the EGFR tyrosine kinase inhibitor AG1478 (AG) but not the MEK inhibitor PD98059 (PD) blocked EGFR phosphorylation by aldosterone (Figure 3G-3I), while ERK1/2 activation was prevented in the presence of both AG and PD. Hence, the MEK/ERK transduction pathway is activated afterward the engagement of EGFR upon aldosterone treatment in our model system. Aldosterone/MR signalling stimulates the activity and expression of NHE-1, which has been involved in tumor cell migration, invasion and metastasis particularly in breast cancer [6-7, 45]. In this regard, we assessed that aldosterone prompts NHE-1 activity in both SkBr3 cells and B-TEC as evaluated by a fluorescent indicator of cytoplasmic pH changes (Figure 4A). In addition, aldosterone up-regulated NHE-1 at both the mRNA and protein levels as determined by real time PCR (Figure 4B) and immunofluorescence studies performed in SkBr3 cells and B-TEC (Figure 4C-4F). Next, the stimulatory effects induced by aldosterone on NHE-1 protein expression were abolished silencing MR (Figure 5) as well as GPER (Figure 6). Collectively, these findings suggest that NHE-1 regulation by aldosterone requires MR along with GPER.

### Aldosterone induces biological responses through both MR and GPER

**Figure 7:**
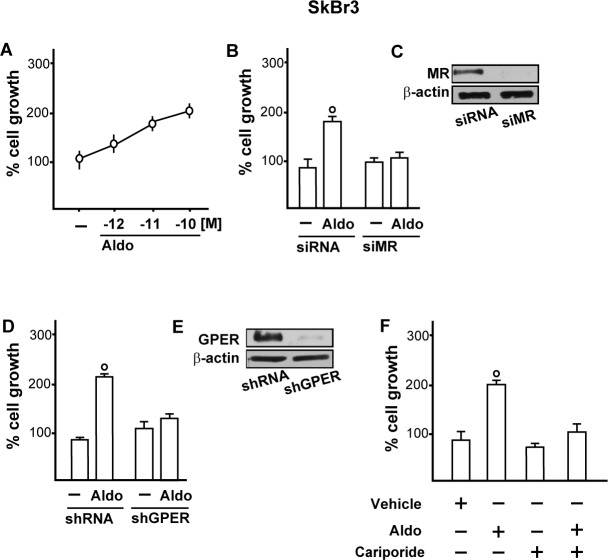
**A.** SkBr3 cell proliferation upon treatment for 5 days with increasing concentrations of Aldosterone (Aldo). Proliferation of SkBr3 cells transfected with siMR **B.**, **C.** and shGPER **D.**, **E.** and treated for 5 days with 10 pM Aldo. SkBr3 cell proliferation stimulated by 10 pM Aldo in the presence of 50 μM Na^+^/H^+^ Exchanger 1 (NHE-1) inhibitor named cariporide **F.**. Values shown are mean ± SD of three independent experiments performed in triplicate. (○) indicates *p* < 0.05 for cells receiving vehicle (−) *versus* Aldo treatment.

**Figure 8:**
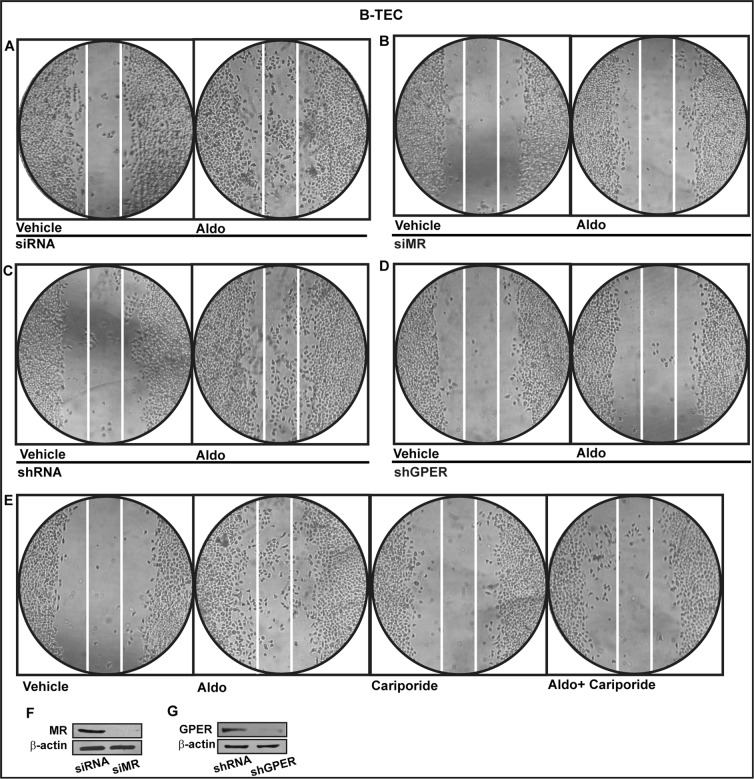
Cell migration in B-TEC transfected for 24 h with siRNA **A.**, siMR **B.**, shRNA **C.** or shGPER **D.** and then treated for 48 hours with ethanol as vehicle or 10 pM Aldosterone (Aldo). **E.** Cell migration stimulated by 10 pM Aldo in B-TEC in the presence of 50 μM Na+/H+ Exchanger 1 (NHE-1) inhibitor cariporide. **F.**, **G.** Efficacy of MR and GPER silencing. Data are representative of three independent experiments performed in triplicate.

Functionally, we studied the role of MR and GPER in the proliferative effects of aldosterone in breast tumor cells as well as in the migration of tumor endothelial cells. Indeed, aldosterone triggered growth effects in SkBr3 cells, as assessed by cell counting (Figure 7A) and evidenced by time-lapse video microscopy (Videos 1-2). Cell proliferation stimulated by 10pM aldosterone was no longer evident silencing MR (Figure 7B-7C) or knocking-down GPER expression (Figure 7D-7E) and using the NHE-1 inhibitor cariporide (Figure 7F). Similar results were obtained using aldosterone concentrations up to 10 nM (data not shown). Furthermore, aldosterone promoted the migration of B-TEC as evidenced by time-lapse video microscopy (Videos 3-4) and scratch assay (Figure 8). The observed aldosterone-induced motility was abrogated silencing MR (Figure 8A, 8B, 8F) or GPER (Figure 8C-8D, 8G) and in the presence of cariporide (Figure 8E). Overall, these results indicate that the functional interaction between MR and GPER is involved in the aforementioned stimulatory action of aldosterone in both SkBr3 cells and B-TEC.

## DISCUSSION

In the present study we provide novel evidence regarding the molecular mechanisms by which GPER may contribute to the biological responses induced by aldosterone in breast cancer cells and breast tumor-derived endothelial cells. In particular, we have demonstrated that aldosterone activates the EGFR/ERK transduction signalling through the classic MR and the involvement of GPER, as evidenced by gene silencing experiments and pharmacological inhibitors. In addition, we have shown that both MR and GPER mediate the aldosterone-induced up-regulation of Na^+^/H^+^ exchanger-1 (NHE-1), a well-known MR target involved in cancer progression [7, 45]. We have also evidenced that aldosterone does not bind to GPER in accordance with previous studies [44], however it triggers the direct interaction between MR and GPER as well as GPER and EGFR. Interestingly, we have determined that both MR and GPER are required for the proliferation and migration of breast cancer cells and B-TEC mediated by NHE-1 upon aldosterone exposure.

Aldosterone elicits important biological effects in several physio-pathological conditions, spanning from electrolyte and fluid homeostasis to the regulation of fibrotic, inflammatory, proliferative and angiogenic responses in cardiovascular, metabolic diseases and cancer [12-13, 46-49]. As it concerns the breast tissue, it has been demonstrated that aldosterone potentiates prolactin stimulation of casein synthesis in pregnant rabbit mammary gland and contributes to mammary gland development and differentiation [50].

The actions exerted by aldosterone mainly occur through the binding to MR, a ligand-inducible transcription factor that belongs to the nuclear receptor superfamily [1]. The enzyme 11β-hydroxysteroid dehydrogenase type II (11βHSD2), which catalyzes the conversion of 11β-hydroxycorticosteroids like cortisol and corticosterone to the respective 11-keto metabolites namely cortisone and 11-dehydrocorticosterone, does allow the aldosterone binding to MR [51]. 11βHSD2 is mainly expressed in mineralcorticoid target tissues like kidney, colon, salivary glands and placenta [51]. In addition, immunohistochemical studies have detected in normal and malignant breast tissues high levels of 11β-HSD2 that co-localize with MR [52]. Previous studies have also evaluated the 11β-HSD2 activity in breast cancer cells, suggesting that this enzyme may play a regulatory role of aldosterone action in breast malignancy [53]. According to the classical model of MR signalling, the interaction between aldosterone and un-liganded receptor promotes the dissociation of the heat shock proteins from MR, which translocates into the nucleus [1]. Then, the aldosterone/MR complex binds to specific response elements located within the regulatory region of target genes, hence resulting in gene expression changes [1]. In addition, aldosterone induces rapid effects through alternate mechanisms including the activation of the EGFR/ERK transduction pathway, as demonstrated in different animal and cell models [3-4]. The existence of aldosterone receptors structurally unrelated to the classic MR paved also the way for analyzing the role of further mediators of the multifaceted action elicited by aldosterone [49].

GPER has been largely demonstrated to mediate estrogenic signalling in a wide number of physio-pathological conditions, including cancer [54-64]. GPER has been also involved in functional responses to aldosterone in various experimental contexts [37-40]. For instance, the ability of aldosterone in activating ERK1/2 in vascular smooth muscle cells and sensitizing the connecting tubule glomerular feedback in afferent arterioles was prevented using both MR and GPER blockers [38-40]. Other studies evidenced that the increase of cardiac vagal tone observed upon aldosterone treatment is abolished in the presence of the GPER antagonist G36 but not using the MR antagonists spironolactone and eplerenone [39]. In rat aortic endothelial cells devoid of MR, the biological effects triggered by aldosterone were mimicked by the GPER agonist G-1 and prevented using pharmacological inhibitors of GPER as well as knocking down its expression [38]. The aforementioned observations suggest that GPER is involved in the effects exerted by aldosterone either through MR or acting as an alternate aldosterone receptor. However, it should be pointed out that diverse controversies argue against the last conclusion, as pharmacologic criteria for GPER to be considered as an aldosterone-responsive receptor are not still adequately fulfilled [41-43]. Indeed, binding studies performed in HEK cells overexpressing GPER (HEK-GPER-1) showed that aldosterone and the MR antagonists, spironolactone and eplerenone, do not compete for specific [^3^H]E2 binding to membrane of HEK-GPER-1 cells [44]. In accordance with these findings, in the present study aldosterone failed to bind to GPER in competition assays based on experimental approaches used in previous investigations in order to characterize the binding properties of GPER ligands [28, 31-32, 34-36]. Worthy, we found that aldosterone stimulates the interaction of GPER with MR and EGFR, thus suggesting a further mechanism through which ligand-activated MR triggers EGFR signalling [49, 65-67]. Nicely supporting the functional cross-talk between MR and GPER, we ascertained that both receptors are required for the aldosterone-induced expression of NHE-1 which is considered as a molecular sensor of MR activation [45]. In this respect, our data are reminiscing of previous findings showing that EGFR and GPER cooperate toward the regulation of NHE-1 function upon aldosterone treatment [40, 66]. Importantly, we found that the stimulatory effects elicited by aldosterone on the proliferation and migration of breast cancer cells and breast tumor-derived endothelial cells are mediated by NHE-1 and involve both GPER and MR. Hence, the current results further extend the well-known action played by NHE-1 toward negative biological features, in particular in breast cancer [7, 68]. In this regard, it is worth mentioning that in tumor metabolic microenvironment characterized by hypoxic-acidic milieu [69], the dysregulation of pH homeostasis mediated by NHE-1 may actually contribute to key steps in tumor progression like increased cell proliferation, loss of cell-cell contact and detachment from the extracellular matrix [68]. In breast cancer cells and breast cancer associated fibroblasts exposed to hypoxia, we have previously assessed that GPER cooperates with hypoxia inducible factor-1 (HIF-1) toward the regulation of vascular endothelial growth factor (VEGF) and tumor angiogenesis [70-73]. Hence, the present findings suggest further mechanisms through which GPER may play a role in the complex adaptive responses to hypoxic-acidic tumor microenvironment. Additionally, our results indicate that GPER contributes to the effects mediated by aldosterone/MR signalling, as evidenced by other ligand-activated steroid receptors [74-75].

Collectively, our findings provide novel insights into the controversial mechanisms through which GPER contributes to aldosterone-mediated signalling. On the basis of our data showing that the functional interaction between MR and GPER triggers certain stimulatory effects exerted by aldosterone, GPER may be considered as a further target within the intricate transduction network activated by aldosterone in particular in breast cancer.

## MATERIALS AND METHODS

### Reagents

Aldosterone (Aldo), 17β-estradiol (E2) and Cariporide were purchased from Sigma Aldrich (Milan, Italy). G15 ((3aS,4R,9bR)-4-(6-bromo-1,3-benzodioxol-5-yl)-3a,4,5,9b-3H-cyclopenta[c]quinolone) was obtained from Tocris Bioscience (distributed by Space, Milan, Italy). Tyrphostin AG1478 (AG) was purchased from DBA (Milan, Itay). PD98059 (PD) was obtained from Calbiochem (DBA, Milan, Italy). All compounds were dissolved in dimethyl sulfoxide (DMSO) except Aldosterone and E2 which were solubilized in ethanol.

### Cell cultures

SkBr3 breast cancer cells were maintained in RPMI-1640 without phenol red, supplemented with 10% fetal bovine serum (FBS) and 100 μg/ml penicillin/streptomycin (Life Technologies, Milan, Italy). Breast tumor-derived endothelial cells (B-TEC) were obtained from human breast carcinomas and characterized as previously described [76]. B-TEC showed constant expression of endothelial markers and increased angiogenic properties, migration and drug resistance in respect to normal microendothelial cells [76-78]. Briefly, specimens were finely minced with scissors and then digested by incubation for 1 h at 37°C in DMEM containing collagenase IV (Sigma Aldrich, Milan, Italy). After washings in medium plus 10% FCS (Life Technologies, Milan, Italy), the cell suspension was forced through a graded series of meshes to separate the cell components from stroma and aggregates. Endothelial cells were isolated from cells suspension using anti-CD105 Ab coupled to magnetic beads, by magnetic cell-sorting using the MACS system (Miltenyi Biotech, Auburn, CA). B-TEC were seeded on collagen-coated flasks (Sigma-Aldrich Srl, Milan, Italy) and cultured in Endothelial Growth Medium (EGM) (Lonza, Milan, Italy), supplemented with 5% FBS (Lonza, Milan, Italy). MCF-7 breast cancer cells were maintained in DMEM F12 supplemented with 10% FBS and 100 μg/mL penicillin/streptomycin (Life Technologies, Milan, Italy). All cell lines were grown in a 37° C HeraCell incubator (ThermoScientific-Heraeus, Milan, Italy) with 5% CO_2_. Cells were switched to medium without serum the day before experiments.

### Saturation curve and scatchard plot analysis

SkBr3 cells were grown in 10-cm cell culture dishes and incubated with increasing concentrations of [2, 4, 6, 7-3H] E2 (89 Ci/mmol; GE Healthcare) or [1, 2, 6, 7-3H] Aldosterone (85 Ci/mmol; Perkinelmer). Cells were then washed with ice-cold phosphate-buffered saline (PBS); after 100% ethanol extraction of cells, radioactivity was measured by liquid scintillation counting. The plot of the bound radioactivity (cpm) *versus* the concentration of the radiotracer (nM) was fitted to the saturation binding curve using Prism GraphPad program (GraphPad Software, San Diego, CA), which was used to calculate the binding dissociation constant (Kd) and binding capacity (Bmax).

### Ligand binding assay

SkBr3 cells were grown in 10-cm cell culture dishes and incubated with 4 nM [2, 4, 6, 7-3H] E2 (89 Ci/mmol; GE Healthcare) or 100 pM [1, 2, 6, 7-3H] Aldosterone (85 Ci/mmol; Perkinelmer) in the presence or absence of increasing concentrations of nonlabeled E2 or aldosterone for 2 hours at 37°C. Cells were then washed with ice-cold PBS; after 100% ethanol extraction of cells, radioactivity was measured by liquid scintillation counting. The displacement of [^3^H]E2 or [^3^H]Aldo binding by the competitors was expressed as a percentage of the maximum specific binding of E2 or Aldo.

### Na^+^/H^+^ Exchanger 1 (NHE-1) activity assay

SkBr3 cells and B-TEC were grown in 10-cm cell culture dishes and then shifted for 24h to medium lacking serum. Then, 4×10^7^ cells/ml were suspended in HEPES buffer solution 1M (Sigma Aldrich, Milan, Italy) and incubated with a membrane-permeable fluorescent indicator for the measurement of cytoplasmic pH namely SPIRO(ISOBENZOFURAN-1(3H),9′-(9H)XANTHENE)-2′,7′-DIPROPANOIC ACID (BCECF-AM) (0,3μM) (Santa Cruz Biotechnology, Milan, Italy) for 30 min at 37°C. Then, cells were washed with HEPES buffer saline and a cell suspension of 3×10^6^ cells/ml was prepared. Fluorescence ratio from the dye was measured using an FLX-800 micro plate fluorimeter (Bio-Tek Instruments, Inc., Winooski, VT, USA).

### Gene expression studies

Total RNA was extracted from cell cultures using the TRIzol commercial kit (Life Technologies, Milan, Italy) according to the manufacturer's protocol. RNA was quantified spectrophotometrically and quality was checked by electrophoresis through agarose gels stained with ethidium bromide. Only samples that were not degraded and showed clear 18 S and 28 S bands under UV light were used for RT-PCR. Total cDNA was synthesized from the RNA by reverse transcription using the murine leukemia virus reverse transcriptase (Life Technologies, Milan, Italy), following the protocol provided by the manufacturer. The expression of selected genes was quantified by real-time PCR using Step One ^(TM)^ sequence detection system (Applied Biosystems Inc, Milan, Italy), following the manufacturer's instructions. Gene-specific primers were designed using Primer Express version 2.0 software (Applied Biosystems. Inc., Milan, Italy) and are as follows: GPER Fwd: 5′-ACACACCTGGGTGGACACAA-3′ and Rev: 5′-GGAGCCAGAAGCCACATCTG-3′; MR Fwd: 5′-GCTTTGATGGTAACTGTGAAGG-3′ and Rev: 5′- TGTGTTGCCCTTCCACTGCT-3′; ERα Fwd: 5′-AGAGGGCATGGTGGAGATCTT-3′ and Rev: 5′-CAAACTCCTCTCCCTGCAGATT-3′; NHE-1 Fwd: 5′:- AAGGACCAGTTCATCATCGC-3′ and Rev:5′- TTCTTCACAGCCAACAGGTC-3′; 18S Fwd: 5′-GGCGTCCCCCAACTTCTTA-3 and Rev: 5′-GGGCATCACAGACCTGTTATT-3′. Assays were performed in triplicate and the RNA expression values were normalized using 18S expression and then calculated as fold induction.

### Gene silencing experiments

For the silencing of GPER expression, cells were plated onto 10-cm dishes and transfected using X-treme GENE 9 DNA Transfection Reagent (Roche Diagnostics, Milan, Italy) for 24 hours with two shRNA and two different shGPER. The silencing of GPER expression was obtained by using constructs which we have previously described and used [79]. For knocking down MR expression, cells were seeded in six-well multidishes and transiently transfected the consecutive day at 50% confluence. For transfection, X-treme GENE 9 DNA Transfection Reagent (Roche Diagnostics, Milan, Italy) was mixed with two small interfering RNAs (siRNA) specific for silencing MR or two siRNA controls (Origene, distributed by Tema Ricerca, Milan, Italy) for 24 hours, prior to treatments.

### Western blot analysis

SkBr3 cells and B-TEC were processed according to a previously described protocol [80-81] to obtain protein lysate that was electrophoresed through a reducing SDS/10% (w/v) polyacrylamide gel, electroblotted onto a nitrocellulose membrane and probed with primary antibodies against MR (PA1594) (Boster Immunoleader, distributed by Tema Ricerca, Milan, Italy), phosphorylated ERK 1/2 (E-4), ERK2 (C-14), EGFR (1005), pEGFR^Tyr 1173^ (sc-12351-R), GPER (N15), ERα (F10) and β-actin (C2), all purchased from DBA (Milan, Italy). Proteins were detected by horseradish peroxidase-linked secondary antibodies (DBA, Milan, Italy) and revealed using the ECL System (GE Healthcare). Precision Plus Protein™ Dual Color Standard (Bio-Rad Laboratories, Milan, Italy) was used to estimate molecular weights and then antigen specificity.

### Coimmunoprecipitation

After stimulation with 10 pM Aldo, SkBr3 breast cancer cells were washed with PBS and lysed using 500 μl RIPA buffer with a mixture of protease inhibitors containing 1.7 mg/ml aprotinin, 1mg/ml leupeptin, 200 mmol/liter phenylmethylsulfonyl fluoride, 200 mmol/liter sodium orthovanadate, and 100 mmol/liter sodium fluoride. Samples were then centrifuged at 13,000 rpm for 10 min, and protein concentrations were determined using Bradford reagent. Protein (250 μg) was then incubated for 2 hours with 900 μl of immunoprecipitation buffer with inhibitors, 2 μg of GPER, MR or EGFR antibody and 20 μl of Protein A/G agarose immunoprecipitation reagent (DBA, Milan, Italy). Samples were then centrifuged at 13,000 rpm for 5 min at 4° C to pellet beads. Pellets were washed four times with 500 μl of PBS and centrifuged at 13,000 rpm for 5 min at 4° C. Supernatants were collected, resuspended in 20 μl RIPA buffer with protease inhibitors, 2X SDS sample buffer (40 mM Tris-HCl; 4% glycerol; 2% SDS) and β-mercaptoethanol and heated to 95° C for 5 min. Samples were then run on 10% SDS-PAGE, transferred to nitrocellulose, and probed with rabbit anti-GPER, rabbit anti-MR or rabbit anti-EGFR antibody. Western blot analysis and ECL detection were performed as described above.

### Immunofluorescence and colocalization studies

50 % confluent cultured SkBr3 cells and B-TEC grown on coverslips were serum deprived and then treated for 8 hours with 10 pM Aldo, as indicated. Where required, cells previously transfected for 24 hours with shGPER or siMR and respective control (as described above) and then treated for 8 hours with 10 pM Aldo. Then cells were fixed in 4% paraformaldehyde, permeabilized with 0.2% Triton X-100, washed three times with PBS and incubated overnight with a goat primary antibody against NHE-1 (C20) (DBA, Milan, Italy). After incubation, the slides were extensively washed with PBS and incubated with 4′,6-diamidino-2-phenylindole dihydrochloride (DAPI), (1:1000), (Sigma-Aldrich, Milan, Italy) and donkey anti-goat IgG-Rhodamine (1:100; purchased from DBA, Milan, Italy). The slides were imaged on the Cytation 3 Cell Imaging Multimode reader (BioTek, Winooski, VT) and analysed using the software Gen5 (BioTek, Winooski, VT).

For colocalization studies SkBr3 cells seeded on chamber slides were serum deprived for 24 hours and then treated for 15 min with 10 pM Aldo. Next, cells were fixed, permeabilized and incubated overnight with anti-rabbit GPER (N15) and anti-mouse MR (H10E4C9F) antibodies (DBA, Milan, Italy) alone and in combination. Slides were then incubated with secondary antibodies (donkey anti-rabbit IgG-Rhodamine, DBA, Milan, Italy) and donkey anti-mouse IgG-Fitch (Alexa Fluor, Life Technologies, Milan, Italy), stained by DAPI and then imaged on the Cytation 3 Cell Imaging Multimode reader (BioTek, Winooski, VT).

### Proliferation assay

For quantitative proliferation assay, SkBr3 cells (1 × 10^5^) were seeded in 24-well plates in regular growth medium. Cells were washed once they had attached and then incubated in medium containing 2.5% charcoal-stripped FBS, transfected for 24 hours, and then treated, as indicated, with transfection and treatments renewed every 2 days. Cells were counted on day 5 using the Countess Automated Cell Counter, as recommended by the manufacturer's protocol (Life Technologies, Milan, Italy).

### Migration assay

Twelve-well plates were coated with 500 μL fibronectin for 2 hours at 37°C (Sigma Aldrich, Milan, Italy). B-TEC were allowed to grow in regular growth medium until they reached a 70% to 80% confluence. Next, cells were incubated in medium containing 2.5% charcoal-stripped FBS and transfected for 24 hours, as indicated. To create a scratch of the cell monolayer, a p200 pipette tip was used. Cells were then washed twice with PBS and treated. The migration assay was evaluated after 48 hours of treatment.

### Time-lapse microscopy

SkBr3 cells and B-TEC (1 × 10^5^) were seeded in 24-well plates in regular growth medium until they reached a 70% to 80% confluence. The culture wells were then incubated in medium containing 2.5% charcoal-stripped FBS, treated and transferred into a time-lapse microscopy platform, equipped with a heated stage chamber (Cytation™3 Cell Imaging Multi-Mode Reader, Biotek, Winooski, VT). Cells were maintained at routine incubation settings (37°C, 5% CO_2_) using temperature and gas controllers. To evaluate cell proliferation and motility, the images were recorded using Cytation 3 Cell Imaging Multimode Reader and the software Gen5 (BioTek, Winooski, VT) in 10 min intervals for 24 hours (cell proliferation) and 10 hours (cell motility). Then, the images were processed as a movie using the software Adobe Creative Cloud Premier Pro CC. Frames collected every 10 minutes are displayed at a rate of 10 frames s-^1^.

### Statistical analysis

Statistical analysis was performed using ANOVA followed by Newman-Keuls' testing to determine differences in means. *p* < 0.05 was considered as statistically significant.

## SUPPLEMENTARY MATERIAL FIGURE AND VIDEOS










