# Supplementary material for: GPER is involved in the stimulatory effects of aldosterone in breast cancer cells and breast tumor-derived endothelial cells
Source: Oncotarget. 2015 Dec 5;7(1):94–111. doi: 10.18632/oncotarget.6475 (PMC4807985; doi:10.18632/oncotarget.6475)
Supplement: Supplementary file 1 [file oncotarget-07-0094-s001.pdf]

GPER is involved in the stimulatory effects of aldosterone in breast cancer cells and breast tumor-derived endothelial cells

Supplementary Material

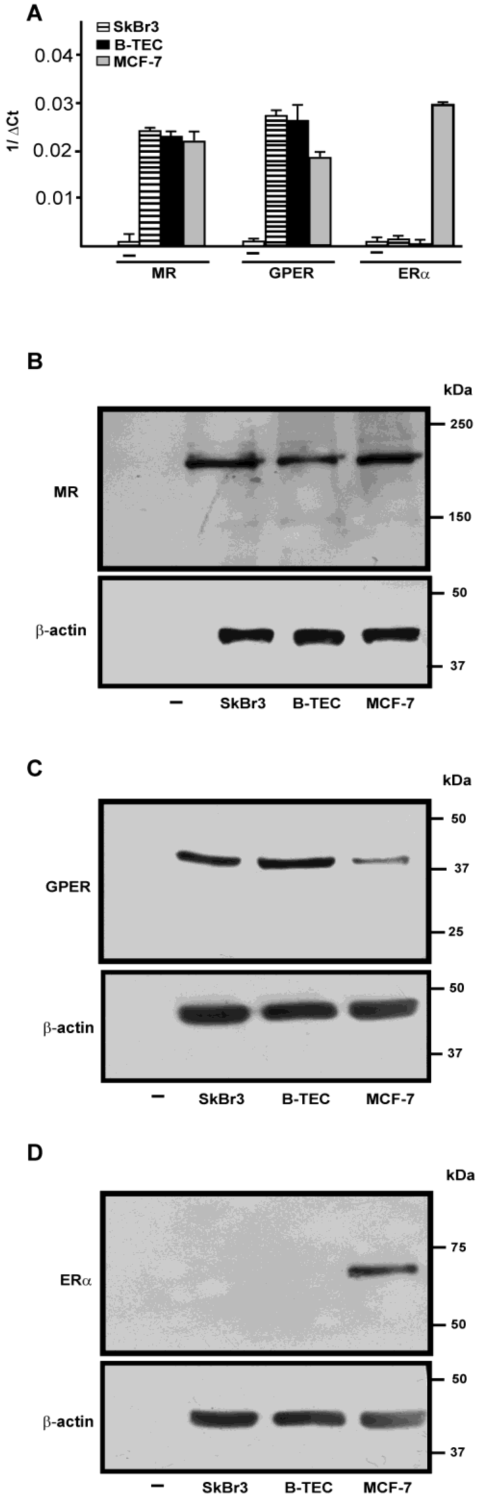

Supplementary Figure 1: (A) mRNA and (B, C and D) protein expression of MR, GPER and ERα in SkBr3, B-TEC and MCF-7 cells. In RNA experiments, PCR amplification in absence of cDNA was used as a negative control (-). In immunoblotting, lysis buffer without proteins was uses as a control in lane 1 (-).

Videos 1-2: Time-lapse video microscopy of live SkBr3 cells treated for 24 hours with vehicle (video 1) or 10 pM aldosterone (video 2). Images were acquired every 10 minutes for 24 hours using Cytation 3 Cell Imaging Multimode Reader and the software Gen5 (BioTek, Winooski, VT). Frames collected every 10 minutes are displayed at a rate of 10 frames s^{-1}.

Videos 3-4: Time-lapse video microscopy of live B-TEC treated with vehicle (video 3) or 10 pM aldosterone (video 4) for 10 hours. Images were acquired every 10 minutes for 10 hours using Cytation 3 Cell Imaging Multimode Reader and the software Gen5 (BioTek, Winooski, VT). Frames collected every 10 minutes are displayed at a rate of 10 frames s^{-1}.
